# Scaling Relationships and Sexual Size Dimorphism Among the Body Parts of *Holotrichia oblita* (Coleoptera: Scarabaeidae)

**DOI:** 10.1002/ece3.71760

**Published:** 2025-07-08

**Authors:** Mengmeng Zhu, Karl J. Niklas, Long Chen, Lin Wang, Yabing Jiao, Peijian Shi

**Affiliations:** ^1^ Institute of Plant Protection Ningxia Academy of Agriculture and Forestry Sciences Yinchuan Ningxia China; ^2^ College of Ecology and Environment Nanjing Forestry University Nanjing Jiangsu China; ^3^ School of Integrative Plant Science Cornell University Ithaca New York USA; ^4^ College of Science Nanjing Forestry University Nanjing Jiangsu China

**Keywords:** body size, *Holotrichia oblita*, scaling relationships, sexual size dimorphism, wing loading

## Abstract

Sexual dimorphism is common among insects. However, whether dimorphism influences the wing loading (i.e., body mass per unit wing area) and scaling relationships among body parts in beetles has seldom been explored. Here, we examined *Holotrichia oblita* (Coleoptera: Scarabaeidae) gender differences in body mass, total hind wing area, leg length, and wing loading, and quantified the scaling relationships between body mass and body length, between head‐and‐prothorax mass and non‐head‐and‐prothorax mass, and between total elytron mass and total hind wing area. Results revealed that (i) females exhibited significantly greater body mass and wing loading, (ii) males showed a relatively larger hind wing area and longer legs (including front, mid, and hind legs), and (iii) scaling analysis demonstrated that the 95% confidence interval of the scaling exponent of body mass versus body length included 3 in both genders. In addition, (iv) the data indicated an isometric relationship between head‐and‐prothorax mass and non‐head‐and‐prothorax mass, and an allometric relationship between total elytron mass and total hind wing area. These results are interpreted to indicate that sexual dimorphism in *H. oblita* likely reflects different selective pressures on gender: the smaller wing loading of males enhances flight maneuverability, potentially aiding in predator avoidance and procuring mates, whereas the larger wing loading and body mass of females provide support for a larger reproductive investment (egg mass). Our study quantifies how sexual dimorphism and tissue‐specific investment alter scaling in beetles. While prior work on leaves and eggs established the role of density heterogeneity, such analyses are scarce in insects, particularly for segmented bodies (head‐thorax‐abdomen) and paired structures (elytra‐wings). By integrating allometry with functional ecology, we reveal how trade‐offs between protection (elytra mass), mobility (wing area), and reproduction (female‐biased size) drive non‐isometric growth, potentially advancing predictions of insect performance under selective pressures.

## Graphical abstract

Females of *Holotrichia oblita* have larger wing loadings and shorter leg lengths compared to males. There are significant allometric relationships between body mass and body length, and between total elytra mass and total hind wing area.

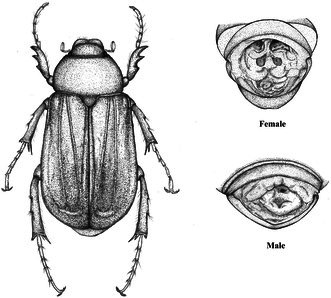

## 1 Introduction

Sexual dimorphism is widespread among metazoans and is commonly observed as differences in body size, morphological traits, and chemical secretions (Coelho 1997; Colgoni and Vamosi 2006; Benítez et al. 2010; Stillwell et al. 2010; Canto et al. 2019; Budečević et al. 2021; Alavi et al. 2022; Shi et al. 2022). In insects, sexual size dimorphism typically favors larger females, whereas cases of male‐biased sexual size dimorphism are relatively uncommon (Fairbairn 1997; Teder and Tammaru 2005). Fecundity selection in females and sexual selection in males are considered the major driving forces behind the evolution of larger body size in insects (Andersson 1994). Larger females generally exhibit enhanced fecundity, enabling them to produce and carry more eggs (Benítez et al. 2010; Allen et al. 2011; Budečević et al. 2021), whereas larger males may gain mating advantages through intragender competition in species with physical contests (Shine 1988; Andersson 1994). The maintenance of size dimorphism likely reflects trade‐offs between opposing pressures; for example, although larger size confers reproductive benefits, it may increase predation risk depending on ecological context (Arak 1988; Schluter et al. 1991; Blanckenhorn 2000; Allen et al. 2011). For instance, larger cicadas typically require longer eclosion times under identical thermal conditions, thereby increasing their risk of predation (Shi et al. 2013, 2022). Similarly, in Lepidopteran larvae, predation risk correlates positively with body size (Remmel and Tammaru 2009). Thus, in systems where agility affects fitness, smaller males may gain advantages by virtue of having greater agility for foraging and finding mates (Zeh et al. 1992; Andersson 1994; Kelly et al. 2008; Shi et al. 2022). These and other selection pressures may drive specific gender adaptations in morphology and resource allocation (Rossi de Gasperis et al. 2017; Budečević et al. 2021; Shi et al. 2022).

The functional traits of insect wings are of particular importance because they influence mobility and flight performance. Although a larger body mass can improve reproductive success and thus fitness, among flying animals, increased body mass typically necessitates a proportionally larger wing area and stronger flight muscles (Coelho 1997). This trade‐off can be quantified using the quotient of body mass and total wing area (i.e., wing loading) (Shi et al. 2015; Shi et al. 2022; Kelly 2020). Studies have shown that lower wing loading is associated with enhanced flight maneuverability in insects (Boiteau and Colpitts 2001; Shi et al. 2015; Kelly 2020). Consequently, sexual size dimorphism often extends to wing morphology (Rossi de Gasperis et al. 2017; Kelly 2020; Ludoški et al. 2023). In the case of the common drone fly, *Eristalis tenax* , adult females have a larger wing area than males, as is the case for two species of underwing moths, *Catocala abacta* and *Catocala brandti* (Alavi et al. 2022). The differences in body mass and wing size between genders indicate that gender may influence wing loading and, consequently, flight performance. For example, selection pressures often target female reproductive traits (e.g., egg production). Consequently, their larger body size increases their wing loading, thereby reducing flight agility compared to males (Benítez et al. 2010; Allen et al. 2011; Alavi et al. 2022). Although larger wing loadings may negatively affect the ability to avoid predators, they may provide an advantage in reproductive investment (Samietz and Köhler 2012).

In beetles, the forewings (elytra) are sclerotized and serve as protective shields, while flight is mediated by membranous hind wings unfolded beneath the elytra (Grimaldi and Engel 2005; Hunt et al. 2007; Johansson et al. 2012; Linz et al. 2016). Although elytra enhance survival in harsh environments, their rigidity increases wing loading, and unfolding the hind wings requires additional energy consumption, thus reducing flight efficiency (Johansson et al. 2012; Goczał et al. 2018). Beetles predominantly rely on legs for locomotion, although they also function in excavation, food manipulation, and intragender combat (Zeh et al. 1992; Arakaki et al. 2004; Weissman et al. 2008; Arnold et al. 2017). Leg morphology plays a crucial role in maneuverability and ecological adaptability and often displays sexual dimorphism (Zeh et al. 1992; Kelly et al. 2008; Weissman et al. 2008; Arnold et al. 2017). For example, female *Glenea cantor* possesses longer legs and specialized bristle structures to grip bark during oviposition, which increases their success rate in egg‐laying (Yan et al. 2024). In addition, sexual selection has led to exaggerated leg traits in males of many beetle species. Enlarged or elongated legs often improve mating success by facilitating male–male competition, enabling males to grasp females during copulation, or enabling males to arrive at mating sites earlier (Zeh et al. 1992; Andersson 1994; Weissman et al. 2008). Male *Acrocinus longimanus* possess disproportionately long forelegs (up to 150 mm), nearly doubling the length of females despite comparable body sizes (Zeh et al. 1992). Similarly, male Jerusalem crickets ( *Stenopelmatus fuscus* ) exhibit longer legs than females, a trait linked to intrasexual competition (Weissman et al. 2008). These characteristics reflect the adaptability of beetles to specific environments.

Resource allocation strategies of insects reflect evolutionary trade‐offs between morphology and ecological function. These patterns are often analyzed through scaling analysis, which quantifies size‐dependent relationships between functionally related biological traits. These relationships are typically described by a power‐law equation taking the form of *Y* = *βX* ^*α*^, where *Y* and *X* are two interdependent variables (e.g., body mass and body length), *α* is the scaling exponent (the slope on a log–log scale), and *β* is the normalization constant (the *y*‐intercept on a log–log scale) (Milla and Reich 2007; Niklas et al. 2007). Mathematically, this implies that $\mathrm{dY}/\mathrm{dX}\propto X^{\alpha -1}$ and $\alpha =\frac{\mathrm{dY}/Y}{\mathrm{dX}/X}$. Therefore, when *α* > 1, the derivative of *Y* with respect to *X* is an increasing function of *X*; when *α* < 1, the derivative of *Y* with respect to *X* is a decreasing function of *X*; and when *α* = 1, the derivative of *Y* with respect to *X* is a constant. These scaling relationships reflect how organisms allocate limited metabolic resources across different body parts. For example, the scaling exponents for insect body dry mass and body length are generally less than 3, indicating that as body length increases, insects tend to become thinner (Schoener 1980; Smock 1980; Gowing and Recher 1984). Ecological gradients further modulate these patterns, for example, high‐latitude species exhibit stouter forms (α closer to 3) to conserve heat, whereas tropical insects often exhibit more slender shapes with scaling exponents less than 3, which presumably enhances cooling and maneuverability (Schoener 1980; Martin et al. 2014). These factors collectively shape the scaling relationship between body dry mass and dry length in insects, reflecting ecological and adaptive strategies.

In addition, the numerical values of scaling exponents often differ between males and females, driven by their distinct reproductive roles and selection pressures (Colgoni and Vamosi 2006; Rossi de Gasperis et al. 2017; Alavi et al. 2022). For example, driven by sexual selection pressure, *Batocera rubus* (Cerambycidae) males show allometry in combat traits (e.g., mandible length, *α* > 1), whereas females typically manifest isometric growth for fecundity (Kawano 2006). Similarly, in the case of *C. abacta* and *C. brandti* , males exhibit allometric growth in wing morphology and function, whereas females mainly exhibit stable scaling relationships, aligning with flight performance demands (Alavi et al. 2022).

**FIGURE 1:**
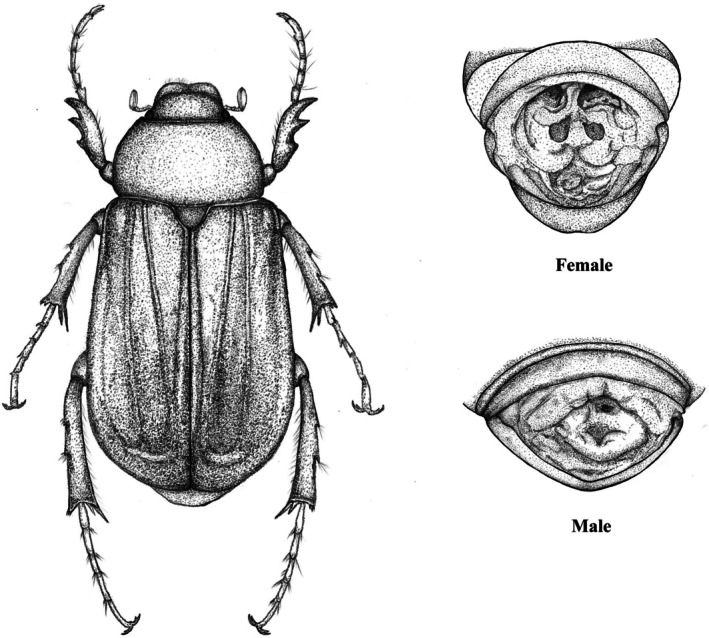
Drawing of the structure of *Holotrichia oblita* and the representation of female and male abdominal tips. When the abdominal tip is opened, two separate patches of sclerite can be seen in a female beetle but are absent in a male beetle.

Although sexual size dimorphism is common in beetles, its influence on wing loading and the scaling relationships among body parts has rarely been systematically investigated. In this study, we focus on the chafer beetle, *Holotrichia oblita*, a prevalent subterranean pest widely distributed in China that causes substantial damage to crops, forests, nurseries, and lawns (Wei et al. 1989). It is convenient for us to collect plenty of adult samples and identify sex via distinct abdominal sclerite patches in females (Chen et al. 2010) (Figure 1). The present study determines (i) whether there are significant differences in body mass, total hind wing area, front leg length, mid leg length, hind leg length, and wing loading between the two genders; (ii) whether there are statistically robust scaling relationships between body mass and body length, between head‐and‐prothorax mass and non‐head‐and‐prothorax mass, and between total elytron mass and total hind wing area.

The scaling relationships between functionally interdependent morphological traits provide critical insights into fundamental constraints governing biomass allocation, biomechanical design, and ecological adaptation in biological systems. While geometric similarity principles predict specific isometric exponents under idealized conditions of uniform density and shape preservation (e.g., mass ∝ volume ∝ length^3^, mass ∝ volume ∝ surface area^3/2^; Thompson 1917), empirical studies across diverse taxa consistently reveal deviations from these expectations (Lin et al. 2018; Shi et al. 2023, 2024). These deviations arise from intrinsic tissue heterogeneity (e.g., variations in material density between protective structures like chitinous exoskeletons and functional organs like membranous wings), developmental trade‐offs, and selective pressures optimizing performance in specific ecological contexts (Niklas 1994a; Lin et al. 2018; Shi et al. 2023, 2024). In beetles, where distinct body compartments serve specialized roles (elytra for protection, hind wings for flight, legs for locomotion, abdomen for reproduction), quantifying the scaling exponents between key traits, such as body mass versus length, mass allocation across functional modules (e.g., head‐prothorax vs. abdomen), and protective investment relative to flight apparatus (elytra mass vs. hind wing area), is essential. Such analyses reveal whether biomass allocation adheres to geometric similarity or reflects allometric strategies shaped by competing selective demands. Investigating these scaling relationships in *H. oblita*, particularly in the context of sexual dimorphism, therefore addresses a significant gap in understanding how morphological integration and developmental constraints underpin functional divergence between genders in ecologically important Coleoptera. This knowledge advances our understanding of fundamental functional morphology and biomass allocation patterns across different organs of arthropods.

## 2 Materials and Methods

### 2.1 Beetle Sampling

A total of 551 adult female and male *H. oblita* (315 females and 236 males) were collected in Nanjing, China, from June to July 2022 (Figure 1) between 21:40–22:40 on the lakeside of Yangshan Lake Park (32°6′24″ N, 118°56′9″ E). The specimens were stored in a box containing mosquito netting to provide nesting sites and host plants during transit (including *Cirsium arvense* var. *integrifolium* , *Prunus × yedoensis*, and *Veronica persica* ). All of the specimens were collected in a spatially homogeneous environment to reduce the potential effects of differences in soil type, host plant species, and microclimate conditions.

### 2.2 Data Acquisition

The fresh body mass (BM) of each beetle was measured using an electronic balance (ME204/02, Mettler Toledo Company, Greifensee, Switzerland; with an accuracy of 0.0001 g). Body length (BL) was measured using a ruler (accuracy 1 mm) defined as the distance from the front of the head to the tip of the abdomen along the midline of the body. Surgical scissors were used to separate each pair of elytra, hind wings, front legs, middle legs, hind legs, and head‐and‐prothorax to measure total elytron mass (EM), head‐and‐prothorax mass (HPM) and the length of the left front leg (FL), left middle leg (ML), and left hind leg (HL). The non‐head‐and‐prothorax mass (NHPM) was defined as BM minus HPM. The total elytron mass per individual was defined as equal to the mass of the two elytra. The hind wings were scanned using a photo scanner (Epson V550, Batam, Indonesia) at 600 dpi resolution. The images were transferred to black‐ and‐white images in .bmp format using Adobe Photoshop CS6 (version 13.0; Adobe, San Jose, CA, USA). Matlab (version ≥ 2009a; MathWorks, Natick, MA, USA) was then used to extract the wing planar coordinates using the M‐file developed by Shi et al. (2018) and Su et al. (2019). The “bilat” function in the “biogeom” package (version 1.3.5) based on the R software (version 4.3.0; R Core Team 2023) was used to calculate total hind wing areas (A). The total wing area per individual was equal to the sum of the wing areas of the two hind wings. The wing load of each beetle (WL) was calculated as the quotient of A and fresh body mass (i.e., WL = A/BM).

### 2.3 Statistical Analyses

The *t* test (Student 1908) was used to determine whether there were significant differences in BM and wing loading (WL) between the two genders at the 0.05 significance level. Correlation analysis was used to evaluate the relationships among BL and FL, ML, HL, as well as A in the two genders.

The power‐law equation, *Y* = *βX* ^*α*^, was used to fit the scaling relationships between any two of the variables of interest (e.g., BM vs. BL), where *Y* and *X* represent any two interdependent variables of interest, *β* is a normalization constant, and *α* is the scaling exponent of the *Y* versus *X* scaling relationship (Niklas 1994a; Niklas et al. 2007). To normalize the data, the power‐law equation was log‐transformed to take the form *y* = *γ* + *αx*, where *y* = log(*Y*), *x* = log(*X*), and *γ* = log(*β*). Reduced major axis protocols were used to estimate the regression and statistical parameters (Niklas 1994a; Quninn and Keough 2002; Smith 2009). The bootstrap percentile method (Efron and Tibshirani 1993; Sandhu et al. 2011) was used to calculate the 95% confidence intervals (CIs) of the slopes of linear log–log regression curves and to test whether the numerical values of any two intercepts or two slopes (between the two genders) differed significantly (Efron and Tibshirani 1993; Sandhu et al. 2011). All statistical analyses were performed using R (R Core Team 2023).

## 3 Results

**FIGURE 2:**
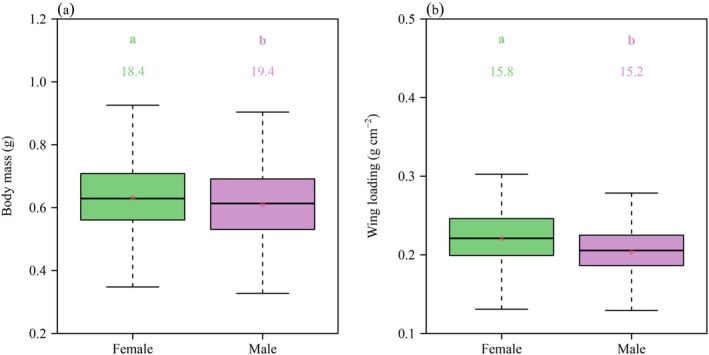
Boxplots of (a) fresh body mass, (b) wing loading. In each panel, the letters on the whiskers show the difference between the two genders (i.e., different letters indicate a significant different between genders at the *α* = 0.05 significance level using *t*‐test); the values at the top of the whiskers represent the coefficients of variation (%) for each sex; the horizontal solid line represents the median value; and the red asterisk represents the mean value. The whiskers extend to the most extreme data point, which is no more than 1.5 times the interquartile range from the box.

**FIGURE 3:**
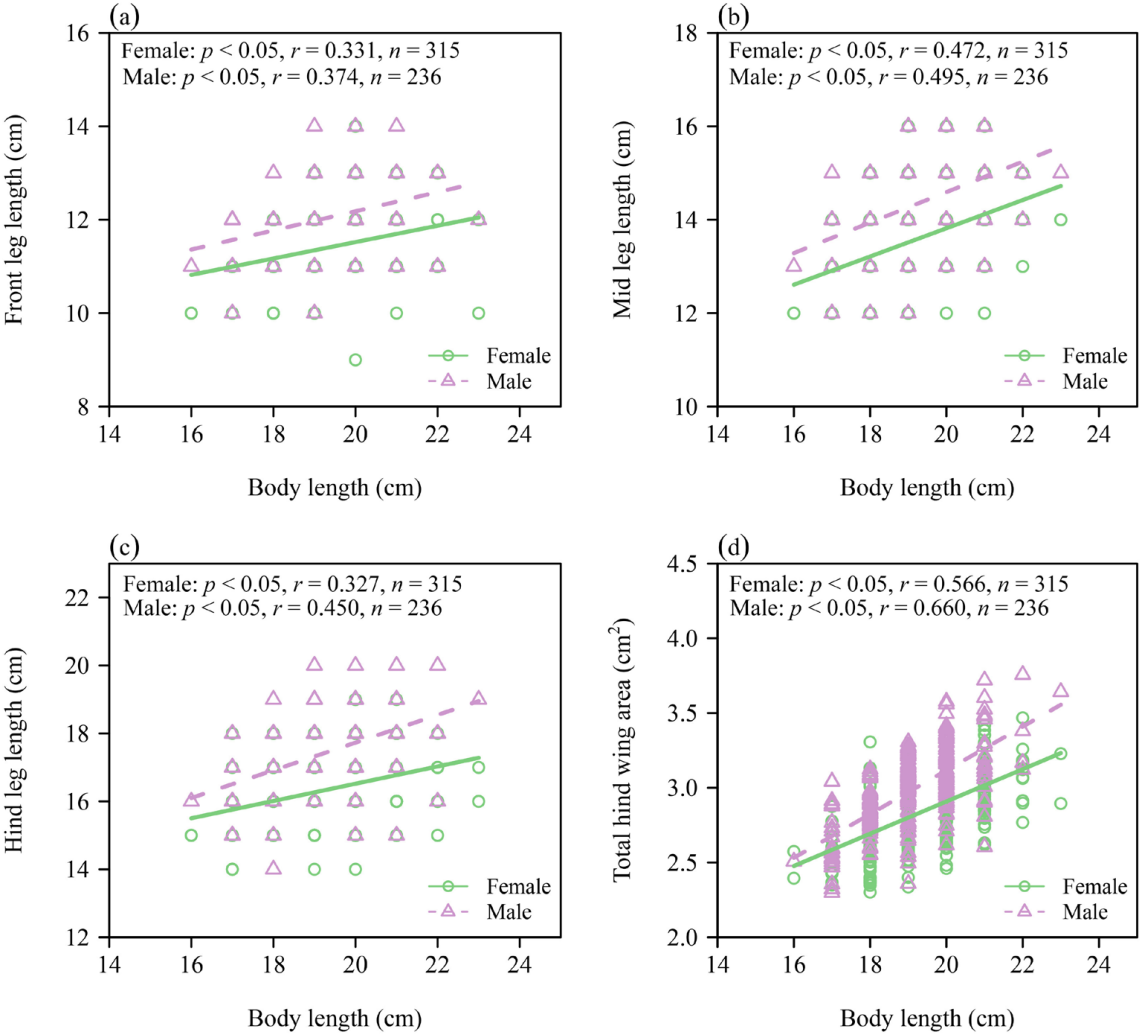
Correlation analysis of the body length and front leg length (a), middle leg length (b), hind leg length (c) total hind wing area (d) for the two genders.

**FIGURE 4:**
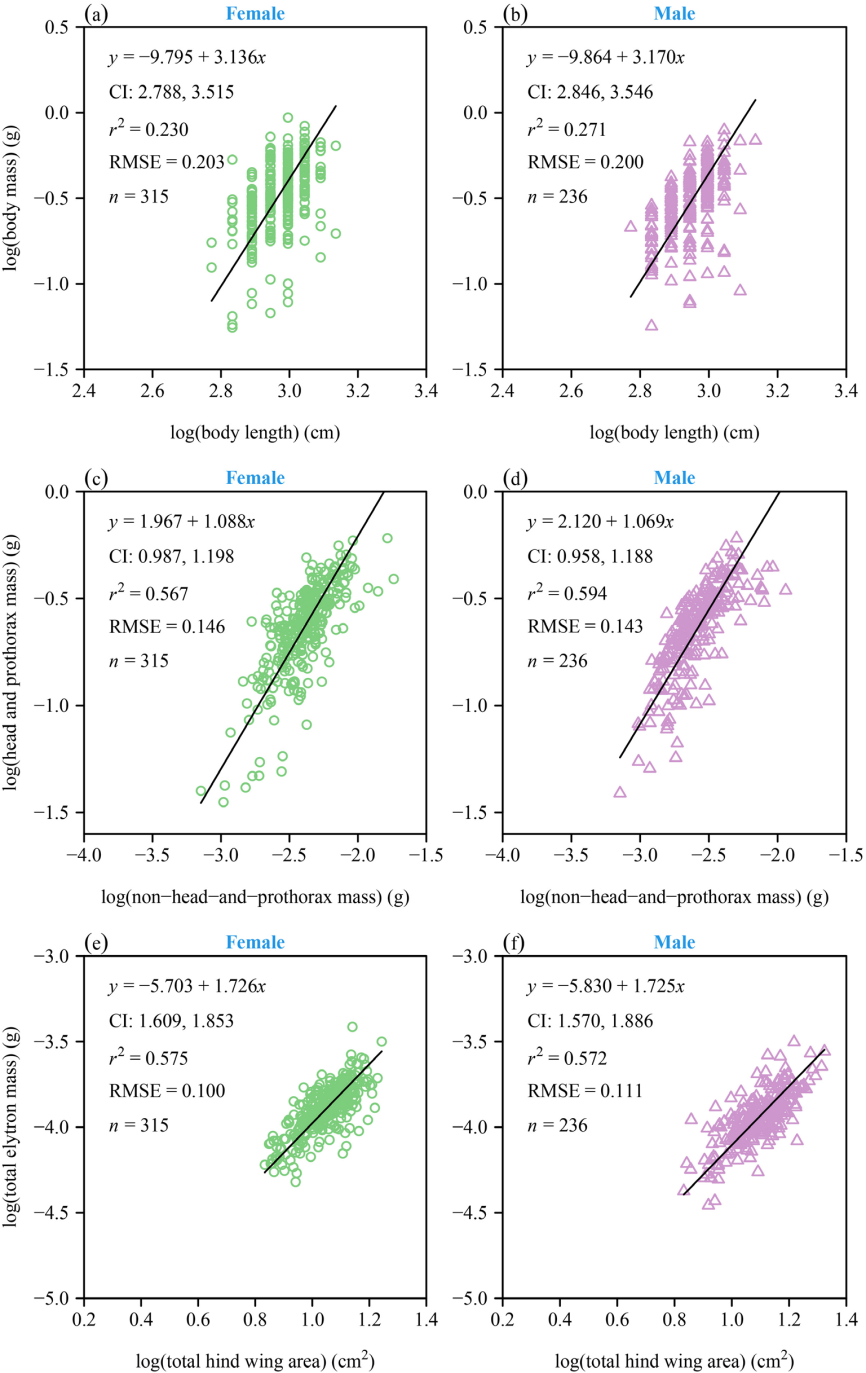
Fitted log–log scaling relationships for body mass versus body length (a, b), head‐and‐prothorax mass versus non‐head‐and‐prothorax mass (c, d), and total elytra mass versus total hind wing area (e, f) for the two genders. The black straight lines are the regression lines; CI is the 95% confidence intervals of the slope; RMSE is the root‐mean‐square error; *r* ^2^ is the coefficient of determination; *n* is the sample size.

There were significant differences in the means of body mass (BM) and wing loading (WL) between the two genders, and the means of BM and WL of females were significantly larger than those of males (*p* < 0.05; Figure 2). Correlation analysis showed that there was a statistically robust positive correlation between body length (BL) and front leg length (FL), middle leg length (ML), hind leg length (HL), and total hind wing area (A) in both female and male beetles. In addition, when BL was approximated as a constant, the FL, ML, HL, and A of males were relatively larger than those of females (Figure 3). The numerical values of the scaling exponents of BM versus BL and total elytron mass (EM) versus A were all significantly greater than unity for both genders (Figure 4). Thus, increases in BL did not keep pace with the increases in BM, and increases in A did not keep pace with the increases in EM. The corresponding 95% CIs of the scaling exponent of head‐and‐prothorax mass (HPM) versus non‐head‐and‐prothorax mass (NHPM) included unity for both genders, indicating that increases in HPM were proportional to the increases in NHPM (Figure 4c,d). Table 1 summarizes the statistical information obtained for *H. oblita* .

**TABLE 1:** Body size measures of *Holotrichia oblita*.

Gender	Body mass (g)	Body length (mm)	Head‐and‐prothorax mass (g)	Non‐head‐and‐ prothorax mass (g)	Total elytron mass (g)	Total hind wing area (cm^2^)	Front leg length (mm)	Mid leg length (mm)	Hind leg length (mm)
Female	0.63 (0.12)^a^	19.54 (1.23)	0.093 (0.018)	0.54 (0.10)	0.021 (0.003)	2.86 (0.23)	11.44 (0.65)	13.68 (0.79)	16.40 (0.96)
Male	0.61 (0.12)	19.14 (1.23)	0.076 (0.016)	0.53 (0.11)	0.019 (0.003)	2.99 (0.27)	12.00 (0.67)	14.31 (0.81)	17.38 (1.11)

^a^ The number in the parentheses represents the standard error. There were 315 females and 236 males.

## 4 Discussion

The data indicate that females have a larger body mass and larger wing loading compared to males. For any given body length, females have relatively shorter front legs, mid legs, and hind legs, as well as a smaller total hind wing area compared to males. The data also indicate that, for both genders, (i) the 95% confidence intervals of the fresh body mass versus body length scaling exponent include 3, and that (ii) an isometric scaling relationship exists between head‐and‐prothorax mass and non‐head‐and‐prothorax mass. In addition, males and females exhibit a statistically significant scaling relationship between total elytron mass and total hind wing area. These findings and their implications are discussed in the following two sections.

### 4.1 Sexual Dimorphism in Body Size, Wing Loading, and Leg Length

Our findings demonstrate that females exhibit a significantly greater body mass than males (Figure 2a), consistent with previously documented trends in insects (Honek 1993; Canto et al. 2019; Budečević et al. 2021). This pattern is generally attributed to fecundity selection because a larger body size enables females to lay more or larger eggs capable of producing more or larger offspring, thereby achieving greater reproductive success (Honek 1993; Reeve and Fairbairn 1999; Colgoni and Vamosi 2006; Allen et al. 2011).

Although total hind wing area increases with body length in both *H. oblita* genders, females consistently displayed smaller hind wing area than males at equivalent body lengths (Figure 3d). This disparity, coupled with the significantly larger wing loading observed in females (Figure 2b), likely indicates that egg‐carrying may impose a constraint on female flight performance (Shi et al. 2022; Ludoški et al. 2023). In contrast, male beetles with relatively lower body mass, lower wing loading, and proportionally larger wings likely possess greater aerial agility, thereby enhancing their ability to meet mates and evade predators (Kawano 2006; Đorđević et al. 2015; Kelly 2020). Moreover, enhanced flight maneuverability and proportionally larger hind wings may compensate for the limited muscular power that accompanies smaller body size in males (Kawano 1995, 2006).

It is important to note that these interpretations should also consider leg length data, given that flight is an energetically demanding activity for beetles (Xie et al. 2021). Gender‐specific resource allocation is also evident in leg length. Although leg length exhibits a positive scaling relationship with body length in both genders, males consistently develop longer legs compared to females of equivalent body length (Figure 3a,c). This trait likely enhances dispersal and intrasexual competition, as smaller‐bodied males with longer legs demonstrate greater movement capacity (Kelly et al. 2008; Arnold et al. 2017) and may use elongated limbs to grasp females during mating (Zeh et al. 1992; Arakaki et al. 2004). In addition, females exhibit larger wing loading and proportionally shorter legs, reflecting the lower mobility investment which may be beneficial to reproduction. For example, in the case of *Stenobothrus lineatus*, individual egg production decreases by 0.36 eggs per day with each meter increase in the average daily activity radius (Samietz and Köhler 2012). These findings are consistent with field observations indicating that, when approached, male beetles typically move faster than females.

### 4.2 Scaling Relationships

The scaling relationship between body fresh mass and body length in both female and male *H oblita* indicates that the 95% confidence intervals of the scaling exponents for both genders include 3 (Figure 4a,b), indicating that the relationship between body mass and body length corresponds to a cubic relationship (*M* ∝ *L* ^3^). This pattern is interpreted to reflect biological constraints on traits such as mechanical stability and growth dynamics, as well as environmental limitations (Schoener 1980; Smock 1980; Martin et al. 2014). These constraints collectively shape the general body form and scaling patterns that are naturally expected and widely observed across diverse taxa (Niklas 1994b).

The scaling relationship between head‐and‐prothorax mass and non‐head‐and‐prothorax mass sheds additional light on the ecology of *H. oblita* . In beetles, these body compartments serve distinct ecological roles: the head is adapted to specific feeding behaviors (Talarico et al. 2011), whereas the thorax, housing the legs and key muscles, is primarily responsible for locomotion and flight (Frantsevich et al. 2014). In turn, the abdomen, containing reproductive organs and nutrient reserves, plays a crucial role in reproduction, providing energy for gamete production (Boggs 1981). This functional divergence between genders might predict gender‐specific allometric growth patterns. For example, females may allocate more resources to abdomen structures and cause the scaling exponents between head‐and‐prothorax mass and non‐head‐and‐prothorax mass to be less than unity (Honek 1993; Rossi de Gasperis et al. 2017). Contrary to expectations, our results reveal an isometric scaling between head‐and‐prothorax mass and non‐head‐and‐prothorax mass (Figure 4c,d). It may reflect a stable and balanced resource allocation strategy during development. This balance suggests a developmental constraint in *H. oblita* in which neither the head‐thorax (feeding and locomotion) nor the abdomen (reproduction) dominates resource investment (Shingleton et al. 2007). Instead, coordinated growth of these functional modules may enhance survival by integrating feeding efficiency and predator evasion strategies (Wainwright 2007).

Both male and female *H. oblita* exhibit a statistically significant scaling relationship between total elytra mass and total hind wing area. The pooled data indicate that the scaling exponent for this relationship exceeds unity such that increases in the total hind wing area do not keep pace with increases in total elytra mass (Figure 4e,f). This result may be explained by the different functional traits of the elytra and hind wings. Elytra are mainly composed of chitin cuticle, which provides protection, whereas hind wings are membranous and composed of softer and more elastic materials (Linz et al. 2016). The difference in mass per unit volume between the elytra and hind wings indicates that, for each unit increase in hind wing area, a disproportionately larger mass investment is required for the elytra. During ontogeny, beetles tend to prioritize resource allocation for strengthening the structure of their elytra, which is closely associated with environmental pressures such as predation, increased risk of physical damage, and water loss in the body. In contrast, slower growth of the hind wing area may indicate a reduced reliance on flight, leading beetles to favor more concealed habitats and short‐distance flight as alternative survival strategies (Evans 1977; Grimaldi and Engel 2005; Hunt et al. 2007; Johansson et al. 2012; Linz et al. 2016). Furthermore, in high‐pressure environments or restricted habitats, enhanced elytra protection may provide a greater survival advantage than flight capability. For example, desert beetles typically possess thickened elytra and exhibit significant wing reduction, a strategy that minimizes water loss and enhances survival in extremely arid environments (Cloudsley‐Thompson 2001).

Finally, males and females exhibit similar scaling exponents among body parts (Figure 4), suggesting that while gender influences body size in *H. oblita* , gender‐specific effects on scaling exponents are minimal. This pattern may arise from shared developmental constraints imposed by their common growth environment and host plants (Teuscher et al. 2009; Stillwell et al. 2010; Silva et al. 2017).

## 5 Conclusions

Significant differences in wing loading and leg length are observed between male and female *H. oblita* . Females have larger wing loading and shorter leg lengths compared to males. We interpret these results to indicate gender‐specific resource allocation strategies wherein males prioritize mating and dispersal‐related traits, whereas females invest more in reproduction. The 95% confidence intervals of the scaling exponents of body mass versus body length for both genders include 3, indicating a cubic relationship consistent with a general Euclidean scaling pattern. An isometric relationship is also observed between head‐and‐prothorax mass and non‐head‐and‐prothorax mass, which permits maintaining the functional effectiveness of the two mass compartments in the beetle body plan. Finally, allometric relationships between total elytra mass and total hind wing area suggest a prioritized investment in rigid elytra over membranous wings. These findings provide additional insights into the beetle body plan and may also provide information for *H. oblita* control.

## Author Contributions

**Mengmeng Zhu:** formal analysis (equal), funding acquisition (lead), writing – original draft (equal). **Karl J. Niklas:** formal analysis (equal), supervision (equal), writing – review and editing (lead). **Long Chen:** formal analysis (equal), investigation (equal), writing – original draft (equal). **Lin Wang:** investigation (equal), writing – review and editing (supporting). **Yabing Jiao:** investigation (equal), writing – review and editing (supporting). **Peijian Shi:** formal analysis (equal), supervision (equal), writing – review and editing (supporting).

## Conflicts of Interest

The authors declare no conflicts of interest.

## Supporting information


Table S1.

## Data Availability

The raw data can be accessed in online Table S1.

## References

[ece371760-bib-0001] Alavi, S. , M. Esfandiari , and M. M. Rabieh . 2022. “Sexual Dimorphism and Allometric Relationships in the Wings of Two Underwing Moths (Lep., Erebidae, Catocala).” North‐Western Journal of Zoology 18, no. 2: 127–134.

[ece371760-bib-0002] Allen, C. E. , B. J. Zwaan , and P. M. Brakefield . 2011. “Evolution of Sexual Dimorphism in the Lepidoptera.” Annual Review of Entomology 56: 445–464. 10.1146/annurev-ento-120709-144828 20822452

[ece371760-bib-0003] Andersson, M. 1994. Sexual Selection. Princeton University Press.

[ece371760-bib-0004] Arak, A. 1988. “Sexual Dimorphism in Body Size: A Model and a Test.” Evolution 42, no. 4: 820–825. 28563875 10.1111/j.1558-5646.1988.tb02501.x

[ece371760-bib-0005] Arakaki, N. , Y. Sadoyama , M. Kishita , et al. 2004. “Mating Behavior of the Scarab Beetle *Dasylepida ishigakiensis* (Coleoptera: Scarabaeidae).” Applied Entomology and Zoology 39, no. 4: 669–674.

[ece371760-bib-0006] Arnold, P. A. , P. Cassey , and C. R. White . 2017. “Functional Traits in Red Flour Beetles: The Dispersal Phenotype Is Associated With Leg Length but Not Body Size nor Metabolic Rate.” Functional Ecology 31, no. 3: 653–661.

[ece371760-bib-0007] Benítez, H. A. , M. A. Vidal , R. Briones , et al. 2010. “Sexual Dimorphism and Morphological Variation in Populations of *Ceroglossus chilensis* (Eschscholtz, 1829) (Coleoptera: Carabidae).” Journal of the Entomological Research Society 12, no. 2: 87–95.

[ece371760-bib-0008] Blanckenhorn, W. U. 2000. “The Evolution of Body Size: What Keeps Organisms Small?” Quarterly Review of Biology 75, no. 4: 385–407. 11125698 10.1086/393620

[ece371760-bib-0009] Boggs, C. L. 1981. “Selection Pressures Affecting Male Nutrient Investment at Mating in Heliconiine Butterflies.” Evolution 35, no. 5: 931–940. 28581061 10.1111/j.1558-5646.1981.tb04959.x

[ece371760-bib-0010] Boiteau, G. , and B. Colpitts . 2001. “Electronic Tags for the Tracking of Insects in Flight: Effect of Weight on Flight Performance of Adult Colorado Potato Beetles.” Entomologia Experimentalis et Applicata 100, no. 2: 187–193.

[ece371760-bib-0011] Budečević, S. , U. Savković , M. Đorđević , L. Vlajnić , and B. Stojković . 2021. “Sexual Dimorphism and Morphological Modularity in *Acanthoscelides obtectus* (Say, 1831) (Coleoptera: Chrysomelidae): A Geometric Morphometric Approach.” Insects 12, no. 4: 350. 33919947 10.3390/insects12040350 PMC8070904

[ece371760-bib-0012] Canto, A. , R. Rodríguez , and E. Reyes‐Novelo . 2019. “Relationship Between the Weights of Seed Beetles of the Genus *Megacerus fåhraeus*, 1839 (Coleoptera: Chrysomelidae: Bruchinae) and Their Host Seeds of the Family Convolvulaceae.” Scientific Reports 9: 8438. 31186456 10.1038/s41598-019-44761-8 PMC6560106

[ece371760-bib-0013] Chen, J. , M. Qu , Q. Ju , et al. 2010. “The Method for Sex Discrimination of Seven Phytophagous Scarab Beetles.” Chinese Journal of Applied Entomology 47, no. 4: 800–805.

[ece371760-bib-0014] Cloudsley‐Thompson, J. L. 2001. “Thermal and Water Relations of Desert Beetles.” Naturwissenschaften 88, no. 11: 447–460. 11771473 10.1007/s001140100256

[ece371760-bib-0015] Coelho, J. R. 1997. “Sexual Size Dimorphism and Flight Behavior in Cicada Killers, *Sphecius speciosus* .” Oikos 79, no. 2: 371–375.

[ece371760-bib-0016] Colgoni, A. , and S. M. Vamosi . 2006. “Sexual Dimorphism and Allometry in Two Seed Beetles (Coleoptera: Bruchidae).” Entomological Science 9, no. 2: 171–179.

[ece371760-bib-0017] Đorđević, M. , U. Savković , J. Lazarević , N. Tucić , and B. Stojković . 2015. “Intergenomic Interactions in Hybrids Between Short‐Lived and Long‐Lived Lines of a Seed Beetle: Analyses of Life History Traits.” Evolutionary Biology 42: 461–472.

[ece371760-bib-0018] Efron, B. , and R. J. Tibshirani . 1993. An Introduction to the Bootstrap. Chapman and Hall.

[ece371760-bib-0019] Evans, M. E. G. 1977. “Locomotion in the Coleoptera Adephaga, Especially Carabidae.” Journal of Zoology 181, no. 2: 189–226.

[ece371760-bib-0020] Fairbairn, D. J. 1997. “Allometry for Sexual Size Dimorphism: Pattern and Process in the Coevolution of Body Size in Males and Females.” Annual Review of Ecology and Systematics 28: 659–687.

[ece371760-bib-0021] Frantsevich, L. , S. Gorb , V. Radchenko , D. Gladun , and A. Polilov . 2014. “Lehr's Fields of Campaniform Sensilla in Beetles (Coleoptera): Functional Morphology. I. General Part and Allometry.” Arthropod Structure & Development 43, no. 6: 523–535. 25240964 10.1016/j.asd.2014.09.001

[ece371760-bib-0022] Goczał, J. , R. Rossa , and A. Tofilski . 2018. “Elytra Reduction May Affect the Evolution of Beetle Hind Wings.” Zoomorphology 137, no. 1: 131–138. 29568156 10.1007/s00435-017-0388-1 PMC5847043

[ece371760-bib-0023] Gowing, G. , and H. F. Recher . 1984. “Length‐Weight Relationships for Invertebrates From Forests in South‐Eastern New South Wales.” Australian Journal of Ecology 9, no. 1: 5–8.

[ece371760-bib-0024] Grimaldi, D. , and M. S. Engel . 2005. Evolution of the Insects. Cambridge University.

[ece371760-bib-0025] Honek, A. 1993. “Intraspecific Variation in Body Size and Fecundity in Insects: A General Relationship.” Oikos 66, no. 3: 483–492.

[ece371760-bib-0026] Hunt, T. , J. Bergsten , Z. Levkaničová , et al. 2007. “A Comprehensive Phylogeny of Beetles Reveals the Evolutionary Origins of a Superradiation.” Science 318: 1913–1916. 18096805 10.1126/science.1146954

[ece371760-bib-0027] Johansson, L. C. , S. Engel , E. Baird , M. Dacke , F. T. Muijres , and A. Hedenström . 2012. “Elytra Boost Lift, but Reduce Aerodynamic Efficiency in Flying Beetles.” Journal of the Royal Society Interface 9, no. 75: 2745–2748. 22593097 10.1098/rsif.2012.0053 PMC3427496

[ece371760-bib-0028] Kawano, K. 1995. “Horn and Wing Allometry and Male Dimorphism in Giant Rhinoceros Beetles (Coleoptera, Scarabaeidae) of Tropical Asia and America.” Annals of the Entomological Society of America 88, no. 1: 92–99.

[ece371760-bib-0029] Kawano, K. 2006. “Sexual Dimorphism and the Making of Oversized Male Characters in Beetles (Coleoptera).” Annals of the Entomological Society of America 99, no. 2: 327–341.

[ece371760-bib-0030] Kelly, C. D. 2020. “Sexual Selection on Size and Shape in Japanese Beetles ( *Popillia japonica* ).” Behavioral Ecology 31, no. 4: 1073–1083.

[ece371760-bib-0031] Kelly, C. D. , L. F. Bussière , and D. T. Gwynne . 2008. “Sexual Selection for Male Mobility in a Giant Insect With Female‐Biased Size Dimorphism.” American Naturalist 172, no. 3: 417–423. 10.1086/589894 18651830

[ece371760-bib-0032] Lin, S. , L. Shao , C. Hui , et al. 2018. “Why Does Not the Leaf Weight‐Area Allometry of Bamboos Follow the 3/2‐Power Law?” Frontiers in Plant Science 9: 583. 29780397 10.3389/fpls.2018.00583 PMC5945892

[ece371760-bib-0033] Linz, D. M. , A. W. Hu , M. I. Sitvarin , and Y. Tomoyasu . 2016. “Functional Value of Elytra Under Various Stresses in the Red Flour Beetle, *Tribolium castaneum* .” Scientific Reports 6: 34813. 27708390 10.1038/srep34813 PMC5052563

[ece371760-bib-0034] Ludoški, J. , L. Francuski , N. Gojković , B. Matić , and V. Milankov . 2023. “Sexual Size and Shape Dimorphism, and Allometric Scaling in the Pupal and Adult Traits of *Eristalis tenax* .” Ecology and Evolution 13, no. 3: e9907. 36937060 10.1002/ece3.9907 PMC10015363

[ece371760-bib-0035] Martin, C. A. , R. Proulx , and P. Magnan . 2014. “The Biogeography of Insects' Length‐Dry Mass Relationships.” Insect Conservation and Diversity 7, no. 5: 413–419.

[ece371760-bib-0036] Milla, R. , and P. B. Reich . 2007. “The Scaling of Leaf Area and Mass: The Cost of Light Interception Increases With Leaf Size.” Proceedings of the Royal Society B 274, no. 1622: 2109–2115. 17591590 10.1098/rspb.2007.0417 PMC2706187

[ece371760-bib-0037] Niklas, K. J. 1994a. Plant Allometry. University of Chicago Press.

[ece371760-bib-0038] Niklas, K. J. 1994b. “The Scaling of Plant and Animal Body Mass, Length, and Diameter.” Evolution 48, no. 1: 44–54. 28567785 10.1111/j.1558-5646.1994.tb01293.x

[ece371760-bib-0039] Niklas, K. J. , E. D. Cobb , Ü. Niinemets , et al. 2007. “‘Diminishing Returns’ in the Scaling of Functional Leaf Traits Across and Within Species Groups.” Proceedings of the National Academy of Sciences of the United States of America 104, no. 21: 8891–8896. 17502616 10.1073/pnas.0701135104 PMC1885598

[ece371760-bib-0040] Quninn, G. P. , and M. J. Keough . 2002. Experimental Design and Data Analysis for Biologists. Cambridge University Press.

[ece371760-bib-0041] R Core Team . 2023. R: A Language and Environment for Statistical Computing. R Foundation for Statistical Computing.

[ece371760-bib-0072] Reeve, J. , and D. Fairbairn . 1999. “Change in Sexual Size Dimorphism as a Correlated Response to Selection on Fecundity.” Heredity 83, no. 6: 697–706. 10651914 10.1046/j.1365-2540.1999.00616.x

[ece371760-bib-0042] Remmel, T. , and T. Tammaru . 2009. “Size‐Dependent Predation Risk in Tree‐Feeding Insects With Different Colouration Strategies: A Field Experiment.” Journal of Animal Ecology 78, no. 5: 973–980. 19493131 10.1111/j.1365-2656.2009.01566.x

[ece371760-bib-0043] Rossi de Gasperis, S. , L. Redolfi De Zan , F. Romiti , et al. 2017. “Sexual Dimorphism and Allometry of Secondary Sexual Character in *Morimus asper* (Coleoptera: Cerambycidae).” Zoomorphology 137: 119–130.

[ece371760-bib-0044] Samietz, J. , and G. Köhler . 2012. “A Fecundity Cost of (Walking) Mobility in an Insect.” Ecology and Evolution 2, no. 11: 2788–2793. 23170213 10.1002/ece3.396 PMC3501630

[ece371760-bib-0045] Sandhu, H. S. , P. Shi , X. Kuang , F. Xue , and F. Ge . 2011. “Applications of the Bootstrap to Insect Physiology.” Florida Entomologist 94, no. 4: 1036–1041.

[ece371760-bib-0046] Schluter, D. , T. D. Price , and L. Rowe . 1991. “Conflicting Selection Pressures and Life History Trade‐Offs.” Proceedings of the Royal Society of London B: Biological Sciences 246, no. 1315: 11–17.

[ece371760-bib-0047] Schoener, T. W. 1980. “Length‐Weight Regressions in Tropical and Temperate Forest‐Understory Insects.” Annals of the Entomological Society of America 73, no. 1: 106–109.

[ece371760-bib-0048] Shi, J. , F. Chen , and M. A. Keena . 2015. “Differences in Wing Morphometrics of *Limantria dispar* (Lepidoptera: Erebidae) Between Population That Vary in Female Flight Capability.” Annals of the Entomological Society of America 108, no. 4: 528–535.

[ece371760-bib-0049] Shi, P. , L. Chen , B. K. Quinn , et al. 2023. “A Simple Way to Calculate the Volume and Surface Area of Avian Eggs.” Annals of the New York Academy of Sciences 1524, no. 1: 118–131. 37106579 10.1111/nyas.15000

[ece371760-bib-0050] Shi, P. , L. Deng , Q. Miao , et al. 2024. “Scaling Relationships of Lamina Mass Per Unit Area, Mean Thickness, and Leaf Bulk Tissue Density Across Nine Diverse Species.” American Journal of Botany 111: e16442. 39644211 10.1002/ajb2.16442

[ece371760-bib-0051] Shi, P. , Y. Jiao , K. J. Niklas , et al. 2022. “Sexual Dimorphism in Body Size and Wing Loading for Three Cicada Species.” Annals of the Entomological Society of America 115, no. 4: 344–351.

[ece371760-bib-0052] Shi, P. , D. A. Ratkowsky , Y. Li , et al. 2018. “General Leaf‐Area Geometric Formula Exists for Plants‐Evidence From the Simpliffed Gielis Equation.” Forests 9, no. 11: 714.

[ece371760-bib-0053] Shi, P. , H. S. Sandhu , and F. Ge . 2013. “Could the Intrinsic Rate of Increase Represent the Fitness in Terrestrial Ectotherms?” Journal of Thermal Biology 38: 148–151.

[ece371760-bib-0054] Shine, R. 1988. “The Evolution of Large Body Size in Females: A Critique of Darwin's ‘Fecundity Advantage’ Model.” American Naturalist 131, no. 1: 124–131.

[ece371760-bib-0055] Shingleton, A. W. , W. A. Frankino , T. Flatt , H. F. Nijhout , and D. J. Emlen . 2007. “Size and Shape: The Developmental Regulation of Static Allometry in Insects.” BioEssays 29, no. 6: 536–548. 17508394 10.1002/bies.20584

[ece371760-bib-0056] Silva, J. A. , A. B. Monteiro , L. F. Maia , and L. D. B. Faria . 2017. “Morphological Traits, Allometric Relationship and Competition of Two Seed‐Feeding Species of Beetles in Infested Pods.” Revista Brasileira de Entomologia 61, no. 3: 243–247.

[ece371760-bib-0057] Smith, R. J. 2009. “Use and Misuse of the Reduced Major Axis for Line‐Fftting.” American Journal of Physical Anthropology 140, no. 3: 476–486. 19425097 10.1002/ajpa.21090

[ece371760-bib-0058] Smock, L. A. 1980. “Relationships Between Body Size and Biomass of Aquatic Insects.” Freshwater Biology 10, no. 4: 375–383.

[ece371760-bib-0059] Stillwell, R. C. , W. U. Blanckenhorn , T. Teder , G. Davidowitz , and C. W. Fox . 2010. “Sex Differences in Phenotypic Plasticity Affect Variation in Sexual Size Dimorphism in Insects: From Physiology to Evolution.” Annual Review of Entomology 55: 227–245. 10.1146/annurev-ento-112408-085500 PMC4760685 19728836

[ece371760-bib-0060] Student . 1908. “The Probable Error of a Mean.” Biometrika 6, no. 1: 1–25.

[ece371760-bib-0061] Su, J. , K. J. Niklas , W. Huang , X. Yu , Y. Yang , and P. Shi . 2019. “Lamina Shape Does Not Correlate With Lamina Surface Area: An Analysis Based on the Simpliffed Gielis Equation.” Global Ecology and Conservation 19: e00666.

[ece371760-bib-0062] Talarico, F. , P. Brandmayr , A. Giglio , et al. 2011. “Morphometry of Eyes, Antennae and Wings in Three Species of *Siagona* (Coleoptera, Carabidae).” ZooKeys 100: 203–204. 10.3897/zookeys.100.1528 PMC3131017 21738413

[ece371760-bib-0063] Teder, T. , and T. Tammaru . 2005. “Sexual Size Dimorphism Within Species Increases With Body Size in Insects.” Oikos 108, no. 2: 321–334.

[ece371760-bib-0064] Teuscher, M. , M. Brändle , V. Traxel , and R. Brandl . 2009. “Allometry Between Leg and Body Length of Insects: Lack of Support for the Size–Grain Hypothesis.” Ecological Entomology 34, no. 6: 718–724.

[ece371760-bib-0065] Thompson, D. W. 1917. On Growth and Form. Cambridge University Press.

[ece371760-bib-0066] Wainwright, P. C. 2007. “Functional Versus Morphological Diversity in Macroevolution.” Annual Review of Ecology and Systematics 38: 381–401.

[ece371760-bib-0067] Wei, H. , Z. Zhang , and M. Wang . 1989. Underground Pests in China. Shanghai Science and Technology Press.

[ece371760-bib-0068] Weissman, D. B. , K. A. Judge , S. C. Williams , D. W. Whitman , and V. F. Lee . 2008. “Small‐Male Mating Advantage in a Species of Jerusalem Cricket (Orthoptera: Stenopelmatinae: Stenopelmatus).” Journal of Orthoptera Research 17, no. 2: 321–332.

[ece371760-bib-0069] Xie, M. H. , Y. Z. Zhong , L. L. Lin , et al. 2021. “Effect of Photoperiod on Longevity, Food Consumption, and Reproduction of *Holotrichia oblita* (Coleoptera: Scarabaeidae).” Environmental Entomology 50, no. 5: 1151–1157. 34240131 10.1093/ee/nvab066

[ece371760-bib-0070] Yan, J. , P. Luo , Y. Wu , et al. 2024. “Morphological and Genetic Differences in Legs of a Polygamous Beetle Between Sexes, *Glenea cantor* (Coleopter: Cerambycidae: Lamiinae).” PLoS One 19, no. 2: e0297365. 38329988 10.1371/journal.pone.0297365 PMC10852293

[ece371760-bib-0071] Zeh, D. W. , J. A. Zeh , and G. Tavakilian . 1992. “Sexual Selection and Sexual Dimorphism in the Harlequin Beetle *Acrocinus longimanus* .” Biotropica 24, no. 1: 86–96.

